# Comparing the fermentation performance of *Escherichia coli *KO11, *Saccharomyces cerevisiae *424A(LNH-ST) and *Zymomonas mobilis *AX101 for cellulosic ethanol production

**DOI:** 10.1186/1754-6834-3-11

**Published:** 2010-05-27

**Authors:** Ming W Lau, Christa Gunawan, Venkatesh Balan, Bruce E Dale

**Affiliations:** 1 Department of Chemical Engineering and Materials Science, DOE Great Lakes Bioenergy Research Center, Michigan State University, 3900 Collins Rd, Lansing, MI 48910, USA

## Abstract

**Background:**

Fermentations using *Escherichia coli *KO11, *Saccharomyces cerevisiae *424A(LNH-ST), and *Zymomonas mobilis *AX101 are compared side-by-side on corn steep liquor (CSL) media and the water extract and enzymatic hydrolysate from ammonia fiber expansion (AFEX)-pretreated corn stover.

**Results:**

The three ethanologens are able produce ethanol from a CSL-supplemented co-fermentation at a metabolic yield, final concentration and rate greater than 0.42 g/g consumed sugars, 40 g/L and 0.7 g/L/h (0-48 h), respectively. Xylose-only fermentation of the tested ethanologenic bacteria are five to eight times faster than 424A(LNH-ST) in the CSL fermentation.

All tested strains grow and co-ferment sugars at 15% w/v solids loading equivalent of ammonia fiber explosion (AFEX)-pretreated corn stover water extract. However, both KO11 and 424A(LNH-ST) exhibit higher growth robustness than AX101. In 18% w/w solids loading lignocellulosic hydrolysate from AFEX pretreatment, complete glucose fermentations can be achieved at a rate greater than 0.77 g/L/h. In contrast to results from fermentation in CSL, *S. cerevisiae *424A(LNH-ST) consumed xylose at the greatest extent and rate in the hydrolysate compared to the bacteria tested.

**Conclusions:**

Our results confirm that glucose fermentations among the tested strains are effective even at high solids loading (18% by weight). However, xylose consumption in the lignocellulosic hydrolysate is the major bottleneck affecting overall yield, titer or rate of the process. In comparison, *Saccharomyces cerevisiae *424A(LNH-ST) is the most relevant strains for industrial production for its ability to ferment both glucose and xylose from undetoxified and unsupplemented hydrolysate from AFEX-pretreated corn stover at high yield.

## Background

Lignocellulosic materials are renewable, abundant and economical carbon sources to potentially substitute for large amounts of petroleum for fuels and chemicals production [1,2]. Ethanol is generally expected to be the first major commercial product of this emerging cellulosic biofuels technology. Bioconversion of fermentable sugars to ethanol is of central importance to this technology [3,4]. Therefore, the development of microbial platforms has been extensively pursued to achieve cost-competitive ethanol yield, titer and productivity [5,6].

Among the ethanologenic strains, *Saccharomyces cerevisiae *[7,8], *Zymomonas mobilis *[9] and *Escherichia coli *[10,11] have been widely investigated and developed for cellulosic ethanol production. The unique advantages of the respective strains were discussed thoroughly in the relevant previous publications. An economically-attractive cellulosic technology almost certainly requires the strain to achieve ethanol yield, titer and rate higher than 90%, 40 g/L (5.1%v/v), 1.0 g/L/h, respectively [12]. The native capacity of the strains is not well-suited to fulfill those requirements for commercial cellulosic ethanol production. Thus, metabolic engineering approaches have been exploited in developing strains to effectively (i) uptake and metabolize pentoses [6,13] and/or (ii) channel the carbon sources for ethanol production [14].

Despite the wealth of publications on strain development, efforts to compare their performance are often hampered by the variations in experimental conditions such as sugar type and concentration, media nutrient levels, initial cell density, feedstock pretreatment selection and detoxification (if applied)[15-17]. Differences in the nature of the pretreatment chemistries and degradation compound profiles strongly influence the performance of a fermenting strain [18]. A systematic and rigorous experimental framework is clearly required to compare the performance of the ethanologenic strains.

In this work, we establish a common platform to obtain comprehensive fermentation parameters using *S. cerevisiae *424A(LNH-ST), *Z. mobilis *AX101 and *E. coli *KO11 as the fermenting strains. In addition, the effect of the water-soluble substances (mainly pretreatment-mediated reaction compounds) from ammonia fiber explosion (AFEX) pretreated corn stover on the growth and fermentation of these three strains is investigated. We also examine the fermentation of enzymatic hydrolysate from AFEX-pretreated corn stover at high solids loading.

## Methods

### AFEX-pretreated corn stover (AFEX-CS)

Corn stover (CS) was obtained from the National Renewable Energy Laboratory (NREL; Colorado, USA), milled and passed through a 4 mm screen. The untreated corn stover consisted of 33.2% cellulose, 22.4% xylan, 3.3% arabinan (analyzed using NREL LAP-002 protocol) and 2.3% protein on a dry weight basis. The error (standard deviation of the triplicate analyzes) of the composition is within 1.5% of the average values. We determined the nitrogen content of the corn stover using a Skalar Primacs SN Total Nitrogen Analyzer (Breda, The Netherlands). The principle behind the nitrogen analysis is based on Dumas method using ethylenediaminetetra-acetic acid as the standards. The nitrogen content was converted protein content by multiplying a factor of 6.25. We used the composition data to determine solids loading during enzymatic hydrolysis. The pretreatment conditions were as follows: temperature at 110-130°C; catalyst loading at 1.0 g anhydrous ammonia to 1.0 g dry corn stover ratio; water loading at 0.6 g water to 1.0 g dry corn stover; and 15 min retention time. Each pretreatment batch contained 150 g corn stover on a dry weight basis. The AFEX apparatus, pretreatment conditions and experiment procedures were as reported [19]. The moisture content after overnight air-drying was about 7% on a total weight basis.

### Microbial strains

Metabolically-engineered ethanologens used in this investigation are *S. cerevisiae *424A(LNH-ST), *Z. mobilis *AX101 and *E. coli *KO11. Strains 424A(LNH-ST) and AX101 were provided by Purdue University and the NREL, respectively. *E. coli *KO11 was obtained from American Type Culture Collection with designated number 55124. Genetic modification and reported fermentation performance were previously reported [9,10,13,20].

### Corn steep liquor (CSL)

FermGold™ CSL (Lot: 154-07) from Cargill Inc (MN, USA) was used as the nitrogen source for fermentation. Technical information from Cargill Inc indicated that FermGold™ CSL contained 48.0-52.0% w/w dissolved solids and 19.5-23.5% w/w total protein. In order to prepare 20% w/v CSL, 200 g of FermGold™ whole CSL was diluted to a total volume of 1.0 L with distilled water and pH was adjusted to 7.0 with reagent grade KOH. The insoluble solids were separated from the liquid by centrifugation at 5000 × g for 30 min. In 1 kg of the 20% CSL mixture, 19.0 ± 0.8 g of dry solids were removed after centrifugation. The 20% w/v CSL was sterile-filtered (0.22 μm) and used for media preparation.

### AFEX-CS wash stream (WS) preparation

AFEX-pretreated CS was washed with distilled water at a ratio of 1 g dry CS to 5 mL of water to produce a water extract at 20% w/v solids loading equivalent. In each batch of washing, distilled water was preheated to 60°-70°C and added to 100 g (dry weight equivalent) of AFEX-CS. The water content of the wetted AFEX-CS was reduced by using an in-house manufactured press. The washing was conducted in three cycles - water-extract from a previous cycle of washing was used for the next cycle of washing. In the final cycle of washing, the moisture content of the washed AFEX-CS was reduced to 77 ± 3% w/w on a total weight basis. The AFEX-CS water extract was used for the fermentation studies.

### AFEX-CS enzymatic hydrolysate 6% w/w glucan loading (18% w/w solids loading)

AFEX-CS was enzymatically-hydrolyzed using both cellulase and hemicellulase commercial mixtures. The cellulase mixture consisted of Spezyme^®^CP [86.7 mL/kg CS; 15 FPU/g cellulose] and Novozyme™ 188 [43.7 mL/kg CS; 32 *p*NPGU/g cellulose]. The hemicellulase mixture was Multifect^® ^Xylanase [12.7 mL/kg CS] and Multifect^® ^Pectinase [8.9 mL/kg CS]. Enzymatic hydrolysis was performed for 96 h at pH 4.8, 50°C and 250 rpm agitation. Phosphate buffer (final concentration 0.05 M) and 12 M HCl at loading 0.02 mL/g dry CS. The activity spectrum of the commercial enzymes used was as reported [21]. Other details were as described previously [22]

### Seed culture preparation

The frozen (-80°C) glycerol stock was transferred to 100 mL liquid media (nitrogen source, 50 g/L total sugar, appropriate buffer and antibiotics) in a 250 mL unbaffled flask. The cells were grown overnight under largely anaerobic conditions at their respective temperatures and initial pH, 150 rpm agitation. Details of culture temperature, initial pH, antibiotics, sugar levels and nitrogen source are as listed in Table 1. These conditions depend on the strain and the type of fermentation for which these seed cultures were prepared. In order to investigate the effect of adaptation of AX101 during seed culture on hydrolysate fermentation, 3% glucan loading of AFEX-CS hydrolysate (pH5.5) was used as seed culture media without nutrient supplementation.

**Table 1:** Seed culture media recipe for the three ethanologenic strains

Strain	Temperature (°C)	Buffer/pH	Antibiotics	Sugars concentration	Nitrogen source
KO11	37	0.1 M MOPS/7.0	50 mg/L Chloramphenicol		
		
AX101	30	0.05 M Phosphate/5.5	30 mg/L Ampicillin	50 g/L for Glucose-only and fermentation 30 g/L +20 g/L for co-fermentation and xylose-only	2.0% w/v corn steep liquor (CSL) for fermentation in CSL fermentation; 5.0 g/L yeast extract + 10.0 g/L peptone for wash stream and AFEX hydrolysate fermentation
		
424A-(LNH-ST)	30	0.05 M Phosphate/5.5	50 mg/L Ampicillin		

Note: yeast extract and peptone was only used for seed culture preparation but was not supplemented to the actual ammonia fiber explosion (AFEX)-corn stover hydrolysate during ethanol fermentation.

### Fermentation using CSL as sole nitrogen source

Fermentations were conducted in pH-controlled fleaker fermentors, as described [23]. Each fleaker (200 mL working volume in 500 mL fleaker) was equipped with a pH probe, a needle to add fluids, a needle for sampling and a magnetic stir bar. A six-position magnetic-stirring plate was placed underneath a water bath to drive the bar at 150 rpm. Water temperature of the water was controlled through a recirculation heater at the respective temperature (Table 1). The fermentation media contained 2% w/v CSL, 100 g/L total sugar, designated buffer and antibiotics. The designated volume of seed culture was centrifuged and the cell pellet was resuspended for an initial OD (600 nm) of 0.5 in the fermentor. Fermentations on glucose only, xylose only and a mixture of glucose and xylose at a mass ratio of 7:3 (co-fermentation) were investigated. Fermentation parameters such as cell density, metabolic ethanol yield, volumetric ethanol productivity and specific ethanol productivity were calculated as previously reported [24].

### Fermentation using AFEX-CS enzymatic hydrolysate (6%w/w glucan loading)

Fermentation of the hydrolysate was conducted in shake flask (70 mL in 250 mL flask) as described [24] based on conditions for seed culture preparation listed in Table 1. Initial cell density was 0.5 OD (600 nm). Hydrolysate of AFEX treated corn stover was fermented without washing, detoxification or nutrient supplementation. Error bars shown in the results are standard deviations of duplicates.

### Microplate cell culture

Fermentations of KO11, AX101 and 424A(LNH-ST) on 0.0%, 7.5% and 15.0% w/v solids-loading-equivalent AFEX-Wash Stream (AFEX-WS) were conducted in a 24-well cell culture microplate (BD Falcon #353047, San Jose, CA). Media was supplemented with 2% w/v CSL, 30 g/L glucose and 20 g/L xylose with appropriate buffer (Table 1). Each well contained 2.0 mL media and a glass bead (6 mm diameter) was added to aid stirring. Seed cultures were prepared as described above and the microplate cell culture was initiated at OD (600 nm) of 0.5. The microplate was sealed and fixed in an incubator shaker (150 rpm) by using a microplate clamp system (Applikon Inc, Springfield, IL). An opening (about 1 mm) was made on the seal to allow carbon dioxide produced to escape. The culture temperatures and pH were as shown in Table 1. After 24 hr, fermentations were stopped and samples were taken. Error bars shown in the results are standard deviations of triplicates.

### Wash stream fermentation

Fermentation was conducted with KO11 using 15.0% w/v solids-loadings-equivalent of AFEX-WS with or without addition of commercial enzymes at loadings described in previous section [22]. The wash stream contains less than 2.5 g/L of monomeric glucose and xylose. YEP (yeast extract peptone; final concentration at 5 g/L yeast extract, 10 g/L paptone), 50 g/L glucose and 25 g/L xylose were added into the media mixtures. Fermentation was conducted at 37°C, pH 7.0, in a 125 mL shake flask with a 50 mL working volume. Initial cell density was at 0.5 OD (600 nm). Error bars shown in the results are standard deviations of duplicates.

### HPLC analysis and cell density measurement

The concentrations of glucose, xylose, ethanol, acetate, formate, lactate, glycerol and xylitol in the fermentation and culture experiments were analyzed using high-performance liquid chromatography (HPLC) with a Biorad Aminex HPX-87 H column (CA, USA). The column temperature was maintained at 60°C and the mobile phase (5 mM H_2_SO_4_) was kept at 0.6 mL/min flow rate. The HPLC system used was as reported [24]. Cell densities were measured using a UV/Vis Spectrophotomer (Beckmann Coulter DU720) at wavelength 600 nm. The absorbance reading was converted to the unit of g dry-wt/L. One unit of absorbance at wavelength 600 nm is equivalent to 0.31, 0.47, 0.33 g dry-wt/L for *Z. mobilis *AX101, *S. cerevisiae *424A(LNH-ST) and *E. coli *KO11, respectively.

## Results

### Fermentations using CSL as nutrients supplement

**Figure 1:**
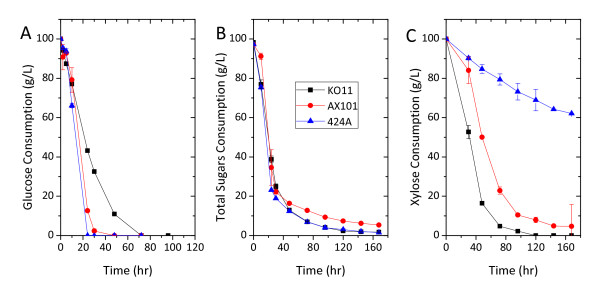
**Fermentation using *Escherichia coli *KO11, *Zymomonas mobilis mobilis *AX101 and *Saccharomyces cerevisiae *424A(LNH-ST) in 2% w/v corn steep liquor with (A) glucose as sole carbon source, (B) glucose and xylose mixture ratio 7:3 and (C) xylose as sole carbon source**. Fermentation was conducted in the fleaker fermentor under largely anaerobic condition and initiated cell density equivalent to 0.5 units OD600 nm. Temperature and pH were controlled at 37°C, 6.8 for KO11 and 30°C, 5.5 for AX101 and 424A(LNH-ST).

Fermentations using 2% w/v CSL as nitrogen source indicated that these three strains effectively produce ethanol from glucose or a mixture of glucose and xylose. During glucose fermentation, the fermentations were completed within 72 h (Figure 1A) and ethanol was produced at concentrations higher than 40 g/L. In particular, 424A(LNH-ST) had the highest rate of glucose utilization at 4.16 g/L/h (Figure 1A). However, an increase in xylose concentration correlated with a decrease of the overall fermentation rate. Overall sugar consumption rates compared between the glucose and xylose fermentation were closest for KO11 followed by AX101 and then 424A(LNH-ST) (Figure 1A and 1C; Table 2). Remarkably, xylose fermentation in 424A(LNH-ST) achieved only 37.9% of xylose consumption after 168 h. Nevertheless, xylose fermentation by 424 A(LNH-ST) was completed when using YEP as the nutrient supplement (Table 2). Specific ethanol productivities of fermentations using the bacteria (AX101 and KO11) as the fermenting strain were at least twice as great as those for 424A(LNH-ST), regardless the type of carbon source (Table 2).

**Table 2:** Results for fermentation using *Escherichia coli *KO11, *Zymomonas mobilis mobilis *AX101 and *Saccharomyces cerevisiae *424A(LNH-ST) in 2% w/w corn steep liquor (CSL) or yeast extract peptone (YEP)

Nutrients Source/sugar Type	Concentration (g/L)	Strain	Sugar consumption (%)*	Metabolic EtOH yield (%)*†	Carbon balance coverage‡	EtOH Concentration*	Volumetric productivity (g/L/hr)†			**Specific productivity (g/L/h/g cell) **^**† **^**0--48 h**
							**Glc 0-24 h**	**Xyl 0-48 h**	**EtOH 0-48 h**	

		KO11	100.0 ± 0.0	87.0 ± 0.9	97.3 ± 1.1	44.3 ± 0.5	-2.37 ± 0.00	N/A	0.79	0.57 ± 0.03
	
CSL/glucose	100	AX101	100.0 ± 0.0	93.2 ± 0.1	95.1 ± 0.1	47.5 ± 0.1	-3.64 ± 0.13	N/A	0.97	0.77 ± 0.07
	
		424A(LHN-ST)	100.0 ± 0.0	85.2 ± 0.5	95.8 ± 0.4	43.5 ± 0.3	-4.16 ± 0.03	N/A	0.87	0.16 ± 0.00

		KO11	98.2 ± 0.5	85.1 ± 1.1	95.9 ± 1.1	41.9 ± 0.8	-2.16 ± 0.07	-0.38 ± 0.03	+0.72 ± 0.01	0.55 ± 0.00
	
CSL/glucose + xylose	70 + 30	AX101	94.5 ± 2.4	88.6 ± 0.0	96.0 ± 0.0	41.5 ± 1.1	-2.43 ± 0.37	-0.29 ± 0.05	+0.77 ± 0.02	0.69 ± 0.02
	
		424A(LHN-ST)	98.4 ± 0.5	82.4 ± 0.5	100.3 ± 0.5	40.2 ± 0.1	-2.76 ± 0.08	-0.39 ± 0.04	+0.73 ± 0.01	0.13 ± 0.00

		KO11	100.0 ± 0.0	85.1 ± 0.0	93.6 ± 0.2	43.1 ± 0.1	N/A	-1.74 ± 0.02	+0.72 ± 0.01	0.54 ± 0.00
	
CSL/Xylose	100	AX101	95.3 ± 0.1	84.9 ± 0.2	97.1 ± 0.1	41.3 ± 0.1	N/A	-1.04 ± 0.01	+0.42 ± 0.00	0.46 ± 0.01
	
		424A(LHN-ST)	37.9 ± 6.5	89.8 ± 1.3	93.2 ± 0.9	18.1 ± 3.2	N/A	-0.32 ± 0.05	+0.09 ± 0.02	0.10 ± 0.00

YEP§/xylose	100	424A(LHN-ST)	100.0 ± 0.0	92.0 ± 0.01	N/A	46.9 ± 0.0	N/A	-1.77 ± 0.02	+0.87 ± 0.01	0.11 ± 0.00

*Time-span for the calculated data: Glc fermentation 0-72 h; Xyl and co-fermentation 0-168 h.

^†^The method to calculate metabolic yields and productivities were as reported in Ref [24].

^‡^Carbon balance covers ethanol, carbon dioxide and all the selected by-products formed during fermentation relative to carbon in consumed sugars. Carbon dioxide production was estimated through the stoichiometric equation for the fermentation of glucose to ethanol - ethanol and carbon dioxide were produced on an equimolar basis.

^§^Yeast extract peptone (YEP), 10 g/L bacto yeast extract +20 g/L bacto peptone.

CSL, corn steep liquor; NA, not applicable.

### Metabolic ethanol yield and byproducts profiles

**Figure 2:**
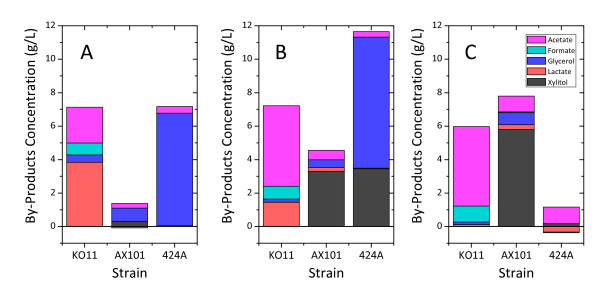
**By-products concentration during (A) glucose fermentation at 72 h, (B) co-fermentation at 168 h and (C) Xylose fermentation at 168 h, in corn steep liquor using *Escherichia coli *KO11, *Zymomonas mobilis mobilis *AX101 and *Saccharomyces cerevisiae *424A(LNH-ST)**.

For AX101 and 424A(LNH-ST), metabolic ethanol yield appeared to decrease in complete fermentation of xylose-containing CSL media (Table 2). Carbon source (glucose or xylose) did not significantly affect the metabolic yield in KO11 fermentation. This trend was also reflected through the profile of targeted byproducts. The total concentrations of the targeted net-byproducts formation in xylose-containing fermentation increased for AX101 and 424A(LNH-ST) compared to glucose-only fermentation, but were essentially unchanged for KO11 (Figure 2).

In xylose-containing fermentation (both xylose only and co-fermentation) by AX101, xylitol is the primary byproduct and it contributed about 70% (58 and 36 mg xylitol/g consumed sugars, in the respective fermentation) of the total measured byproducts. In fermentation by 424A(LNH-ST), net productions of 67 and 81 mg glycerol/g total consumed sugar(s) were observed in glucose and co-fermentation, respectively (Figure 2A and 2B). In addition, xylitol production during co-fermentation contributed to the lower metabolic yield observed compared to glucose-only fermentation.

Organic acids were identified as the predominant group of byproducts from fermentation by KO11 (Figure 2). Although total concentrations of the targeted byproducts were at 6.0-6.5 g/L regardless of carbon source, the byproduct profile varied substantially. While acetate formation increased from 21.4 (glucose-only) to 47.5 mg/g consumed sugar (xylose-only), lactate production diminished during xylose-only fermentation. Of all fermentations, glucose fermentation by AX101 achieved the highest metabolic yield (Table 2) and lowest targeted byproduct formation.

### Fermentation using AFEX-CS wash stream

**Figure 3:**
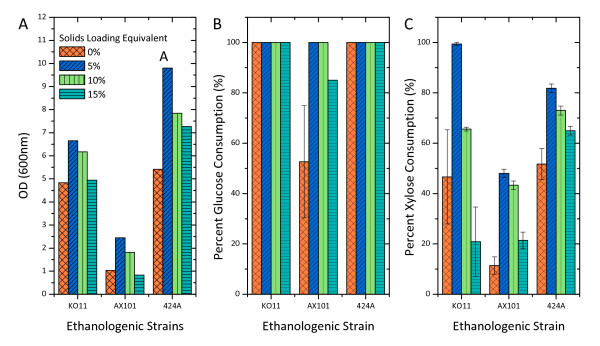
**The effect of water-soluble compounds from ammonia fiber explosion (AFEX)-treated corn stover on (A) cell growth, (B) percent glucose consumption and (C) percent xylose consumption after 24 h fermentation**. The experiments were conducted in 24 wells plate at 2.0 mL working volume under largely anaerobic condition. The initial concentration of glucose, xylose and cell density was 3 g/L, 20 g/L and 0.5 unit of OD600 nm, respectively. The fermentation media was supplemented with 2.5 g/L yeast extract and 5 g/L peptone.

AFEX-CS wash stream was used to provide a representative compound profile found in the pretreated biomass without the involvement of enzymatic hydrolysis. Fermentations by these three strains exhibited similar patterns; in that moderate levels of AFEX-CS wash stream improved cell growth but the degree of improvement decreased as the strength of the wash stream increased. However, a greater cell density was achieved in most of the wash stream-containing fermentations relative to the control (YEP with no wash stream; Figure 3A).

The rate of xylose fermentation correlated well with the cell growth pattern (Figure 3).

KO11 consumed xylose completely at the highest rate (close to 20 g/L/h) at 5% w/v solids loading equivalent of wash stream. However, the rate decreased substantially as the solids loading equivalent increased. In the highest tested solids loading, 424A(LNH-ST) had the greatest xylose consumption rate (12.8 g/L/h) followed by KO11 and AX101.

Although able to ferment at the highest specific rate (g/h/g cells), AX101 consumed both sugars at the lowest volumetric rate. AX101 also appeared to have the lowest tolerance toward water-soluble compounds in AFEX-CS. The cell density of AX101 at 24 h decreased by 66% when the solids loading was increased from 5% to 15% w/w (Figure 3A). This decrease for both 424 A(LNH-ST) and KO11 was 26%.

**Figure 4:**
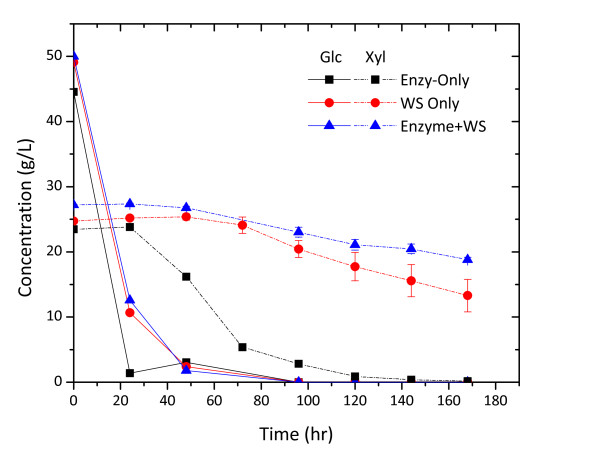
**Fermentation using *Escherichia coli *KO11 on yeast extract peptone (5 g/L yeast extract + 10 g/L peptone) media supplemented with commercial enzymes, 15% solids loading equivalent of ammonia fiber explosion (AFEX)-corn stover wash stream (WS) or a combination of commercial enzymes and the WS**. Fermentation was conducted at 37°C, pH 7.0 (adjusted during fermentation) and was initiated at 0.5 OD600 nm.

The effect of water soluble degradation compounds from AFEX-CS at 15% w/v solids loading equivalent on glucose fermentation was practically negligible for KO11 (Figure 4). However, these compounds were shown to be rather inhibitory toward xylose fermentation (Figure 4). The xylose consumption rate within 96 h in wash stream fermentation was five times lower than that of the control experiment.

#### Fermentation using AFEX-CS hydrolysate (18% w/w solids loading) and AFEX-CS WS

**Figure 5:**
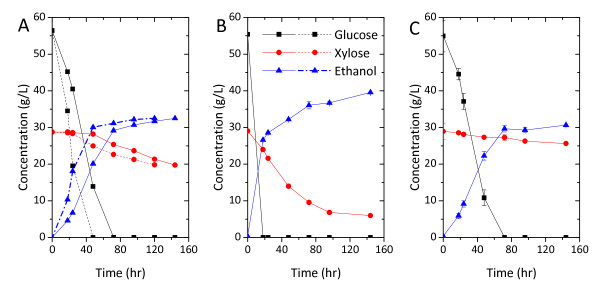
**Fermentation using (A) *Zymomonas mobilis mobilis *AX101, (B) *Saccharomyces cerevisiae *424A(LNH-ST) and (C) *Escherichia coli *KO11, in enzymatic hydrolysate from 6.0% glucan loading of ammonia fiber explosion (AFEX)-pretreated corn stover (CS)**. Fermentation was conducted under largely anaerobic condition and initiated at cell density equivalent to 0.5 unit OD600 nm temperature and pH were controlled at 37°C, 6.8 for KO11 and 30°C, 5.5 for AX101 and 424A(LNH-ST). Solid lines: Seed culture in yeast extract peptone; dotted lines: seed culture in 3% glucan loading of AFEX-CS hydrolysate.

All tested strains were able to grow and completely consume glucose on the AFEX-CS hydrolysate without washing of the pretreated biomass, nutrient supplementation or detoxification (Figure 5). Similar to co-fermentation in CSL (Figure 1B, Table 3), xylose fermentation was considerably slower than glucose fermentation. In the hydrolysate fermentation, xylose fermentation from the tested bacteria (AX101 and KO11) was very poor; less than 20% of the total xylose was consumed (Figure 5A and 5C; Table 3). Hence, xylose fermentation became the bottleneck for yield, concentration and rate for the bacteria. However, nearly complete xylose consumption was achieved in 424A(LNH-ST) fermentation at a metabolic yield of 0.47 g ethanol/g consumed sugars.

**Table 3:** Qualitative summary of the relative fermentation performance of *Zymomonas mobilis mobilis *AX101, *Saccharomyces cerevisiae *424A(LNH-ST) and *Escherichia coli*

Fermentation	Parameters	AX101	424A	KO11
Glucose Consumption	In corn steep liquor (CSL)	Very fast	Very fast	Fast
	
	In lignocellulosic hydrolysate	Average	Very fast	Average

Xylose Consumption	In CSL co-fermentation	Average	Very slow	Fast
	
	In lignocellulosic hydrolysate	Very slow	Average	Very slow

Nutrient Requirement	Glucose-only	Low	Low	Low
	
	Co-Fermentation	Low	Low	Low
	
	Xylose-only	Average	High	Low

Growth Robustness		Average	Very High	High

Metabolic Yield		Very High	High	High

KO11

## Discussion

### Rationale behind the platform for comparison

In this work, we first compared glucose, xylose and co-fermentation in the CSL, followed by co-fermentations on AFEX-CS enzymatic hydrolysate to elucidate its effects on microbial growth pattern and xylose utilization. Fermentations using CSL reveal fermentation performance of respective ethanologens without the interference from degradation products from the pretreated biomass. CSL has also been regarded as a economical nitrogen source in large scale fermentation [25]. Lignocellulosic hydrolysate from AFEX-CS without washing, detoxification and supplementation provided the actual lignocellulosic sugar media for cellulosic ethanol production. This investigation platform would enable us to evaluate the strains based on their intrinsic fermentation ability and robustness for industrial applications.

### Intrinsic fermentation performance of *E. coli *KO11, *Z. mobilis *AX101 and *S. cerevisiae *424A(LNH-ST) in CSL media

*E. coli *KO11, *Z. mobilis *AX101 and *S. cerevisiae *424A(LNH-ST) were able to produce ethanol with a metabolic yield between 82.4-93.2% of theoretical maximum (Table 2) in both glucose and co-fermentation at final concentrations of 40 g/L or higher, at a rate over 0.72 g/L/h (0-48 h). These parameters are comparable to those projected to be necessary for a viable cellulosic ethanol industry [12].

Regardless of media used, fermentations with higher glucose to xylose ratios yielded better results in term of ethanol yield, concentration and rate. Pentose-only fermentation in naturally-occurring xylose-metabolizing strain, such as *E. coli*, has proven to be more difficult than hexose fermentation. One proven cause is the lack of precursors to synthesize products derived from 2-ketoglutarate[26]. In the heterologous pentose metabolic system, additional issues associated with pentose transport [27] and redox balance [6,8] are also reported as potential bottlenecks for xylose-to-ethanol bioconversion.

### Xylose fermentation in AFEX-CS hydrolysate

In this report, xylose fermentation in lignocellulosic hydrolysate is shown to be substantially more challenging relative to co-fermentation in CSL. Furthermore, xylose consumption in the bacterial fermentations was considerably weaker than in 424A(LNH-ST) fermentation. The selective inhibition on the xylose fermentations, presumably from degradation products from pretreated biomass, is not well understood. Hence, improved fundamental understanding of the inhibitory mechanism selectively targets xylose fermentations could help alleviate this crucial process bottleneck.

### Relative strengths and weaknesses of the individual ethanologenic strains

KO11 is able to tolerate a relatively high concentration of AFEX-CS degradation compounds and produces ethanol at a high metabolic yield and rate. Nevertheless, xylose utilization in degradation compound-containing media (high solids loading) is severely affected **(**Figure 5). AX101 is an excellent ethanologenic strain due to its superior metabolic yield and glucose fermentation rate (Table 3). However, the growth robustness of this strain in the media containing degradation products is the lowest among the tested strain. *S. cerevisiae *424A(LNH-ST) is highly robust and able to ferment both glucose and xylose to ethanol reasonably well (greater than 85% of ethanol yield), even at high solids loading (Figure 5).

By comparing these three strains, *S. cerevisiae *424A(LNH-ST) appears to be the most relevant strain for industrial production due to the overall ethanol yield, titer and rate achieved by this strain in undetoxified and unsupplemented AFEX-CS hdyrolysate.

## Conclusions

The intrinsic fermentation performance of all tested ethanologenic strains can fulfill the basic requirements for commercial cellulosic ethanol production. The bacterial metabolic pathway (KO11 and AX101) is more effective at fermenting ethanol from consumed sugars relative to the yeast (424A[LNH-ST]) pathway. However, xylose fermentation is selectively affected during fermentation of pretreated corn stover hydrolysate; the ability to consume xylose in lignocellulosic hydrolysate is the determining factor that dictates overall process yield and concentration. In this regard, *S. cerevisiae *424A(LNH-ST) has shown the highest xylose consumption extent and rate among tested ethanologens.

## Abbreviations

AFEX: ammonia fiber expansion; AFEX-WS: AFEX-CS wash stream; CS: corn stover; CSL: corn steep liquor; NREL: National Renewable Energy Laboratory; YEP: yeast extract peptone.

## Competing interests

The authors declare that they have no competing interests.

## Authors' contributions

MWL designed and carried out experiments, analyzed results and wrote the manuscript. CG carried out experiments and analyzed results. VB and BED analyzed results and reviewed the manuscript. All authors have read and approved the final manuscript.
